# Identification of the Transcriptional Biomarkers Panel Linked to Pathological Remodelling of the Eye Tissues in Various HD Mouse Models

**DOI:** 10.3390/cells11101675

**Published:** 2022-05-18

**Authors:** Iwona Mazur-Michałek, Marcin Ruciński, Mateusz Sowiński, Paulina Pietras, Marta Leśniczak-Staszak, Witold Szaflarski, Mark Isalan, Michal Mielcarek

**Affiliations:** 1 Department of Histology and Embryology, Poznan University of Medical Sciences, 61-701 Poznan, Poland; imazur.michalek@gmail.com (I.M.-M.); marcinruc78@gmail.com (M.R.); matsow@gmail.com (M.S.); paulinapietras29@gmail.com (P.P.); marta.m.lesniczak@gmail.com (M.L.-S.); witold.szaflarski@gmail.com (W.S.); 2 Department of Life Sciences, Imperial College London, Exhibition Road, London SW7 2AZ, UK; m.isalan@imperial.ac.uk; 3 Imperial College Centre for Synthetic Biology, Imperial College London, London SW7 2AZ, UK

**Keywords:** Huntington’s disease, ocular neurodegeneration, retina, transcriptional deregulation, biomarkers, mouse models

## Abstract

Ocular abnormalities are becoming associated with a spectrum of pathological events in various neurodegenerative diseases. Huntington’s disease (HD) is just such an example of a fatal neurological disorder, where mutated genes (CAG trinucleotide expansions in the *Huntingtin* gene) have widespread expression, leading to the production of mutant Huntingtin (mHTT) protein. It is well known that mutant HTT protein is prone to form toxic aggregates, which are a typical pathological feature, along with global transcriptome alterations. In this study, we employed well-established quantitative methods such as Affymetrix arrays and quantitative PCR (qPCR) to identify a set of transcriptional biomarkers that will track HD progression in three well-established mouse models: R6/2, R6/1, and *Hdh*Q150. Our array analysis revealed significantly deregulated networks that are related to visual processes and muscle contractions. Furthermore, our targeted quantitative analysis identified a panel of biomarkers with some being dysregulated even at the presymptomatic stage of the disease, e.g., *Opn1mw*, *Opn1sw*, and *Pfkfb2*. Some of the deregulated genes identified in this study have been linked to other genetic ocular disorders such as: GNAT2, a source of achromatopsia, and REEP6, linked to *Retinitis pigmentosa.* It may thus be a useful platform for preclinical evaluations of therapeutic interventions.

## 1. Introduction

Chronic and progressive neurodegenerative diseases such as Alzheimer’s disease (AD), Parkinson’s disease (PD), and Huntington’s disease (HD) have been associated not only with widespread brain pathology but also with peculiar ocular abnormalities [1]. In fact, the eye is a multi-cellular organ that can be recognised as a window to the brain [2]. The strong link between the eye and the central nervous system has already been found to have consequences in the neurodegenerative pathological conditions where visual manifestations precede central symptoms [3]. The list of such neurodegenerative disorders includes Huntington’s disease: this is a fatal, inherited neurodegenerative disease caused by CAG triplet expansions within exon 1 of the huntingtin gene (*HTT*) [4]. Such triplet expansions translate into a polyglutamine stretches (polyQ) within HTT protein, leading to alterations in its biochemical and structural properties [5,6]. In humans, HD is characterised by a broad spectrum of symptoms including motor dysfunction, cognitive decline, and progressive dementia, with the first abnormalities typically occurring in midlife [6]. In addition, a recent cohort study revealed a number of co-existing non-psychiatric comorbidities in HD gene carriers. The study identified six clusters of comorbid conditions in HD patients: musculoskeletal, allergies, cardiovascular, gastrointestinal, thyroid, and ophthalmologic [7]. Since Huntingtin transcripts can be found in virtually all somatic tissues [8] and during embryonic development [9], it has become apparent that the HD-related pathological abnormalities are not restricted only to the central nervous system, as was initially anticipated [10]. The widespread peripheral HD pathology has been extensively described in various HD mouse models and in HD patients (for a review see [10,11]). Indeed, we have identified a spectrum of pathological events leading to HD-related cardiomyopathy in various HD mouse models [12,13,14], which is likely driven by the functions of intrinsic mutant HTT in the heart [15]. Another example of peripheral abnormalities is the skeletal muscle malfunction in HD mouse models [16,17] and in human gene carriers (for a review see [18]). Interestingly, the HD skeletal muscle pathology can be delayed by a small molecule in the presence of ongoing CNS degeneration [19].

It has recently become apparent that eye tissue may also be affected by HD-related pathology. It has been shown that mutant HTT is expressed in the retina of two HD-fragment mouse models, namely R6/2 and R6/1. In addition, the mutant HTT aggregates load progressively and accumulate throughout three neuronal layers of retina [20]. Consistently, mutant Huntingtin aggregates have been found in the main neuronal population of cells, e.g., cone and rod photoreceptors, bipolar and amacrine cells, as well as in ganglion cells [20]. On the physiological level, an analysis of electroretinograms (ERG) revealed severe retinal dysfunction, with an apparent reduction of both a- and b-waves at maximal intensities, in symptomatic R6/1 mice. These functional deficits were also observed in the same study in the R6/2 mouse model, which suffered progressive abnormalities in visual capabilities at the pre-symptomatic stage (4 weeks of age) [20]. A more recent study of retinal pathological remodelling in symptomatic R6/1 mice, also observed by ERG a severe and progressive deficit in cone response. In addition, there was an increased gliosis of Muller cells and the presence of ectopic rod photoreceptor terminals [21]. Consequently, the pathological retina remodelling in symptomatic R6/1 mice was also observed in the downstream neuronal cell population, in the rod and cone bipolar cells [21].

The early studies in human HD carriers revealed slower tracking eye movements and saccades as the most prominent abnormalities of the oculomotor function (reviewed in [22]). Moreover, other ophthalmic parameters have been shown to be also affected in HD carriers. A study that used spectral-domain optical coherence tomography to measure peripapillary retinal nerve fiber layer (pRNFL) thickness, and the thickness and volume of retinal layers in 15 HD patients, revealed a significant pathological remodelling of the retina. Several features such as temporal and macular retinal nerve fiber layers, the inner plexiform layer, retinal pigment epithelium, and outer macular volume, were found to be significantly lower in HD, in comparison to age-matched controls. However, the outer nuclear layer and outer retinal layer thickness were significantly increased [23].

Although a number of retina-related pathological abnormalities have already been identified in preclinical studies, using a variety of HD mouse models and in human clinical studies, there is an urgent need to establish a panel of quantifiable biomarkers for these processes. This can be achieved by transcription profiling. Hence, our study aims to identify transcriptional dysregulation of the oculomotor transcriptome, in various HD mouse models, using an Affymetrix array analysis, followed by quantitative PCR.

## 2. Material and Methods

### 2.1. Mouse Maintenance and Genotyping

All HD mouse lines were purchased from Jackson Laboratories (US). The R6/2, R6/1, and *Hdh*Q150 HD mouse lines were bred and genotyped as previously described [14,24,25]. The cumulative CAG counts for all time-points were as follows: R6/2 (162 ± 2.8 SD); R6/1 (131 ± 3.1 SD; and *Hdh*Q150 homozygote (165 ± 1.7 SD). All experimental procedures were conducted under a project license from the Home Office, UK, and approved by the Animal Welfare and Ethical Review Body of Imperial College London. Experimental groups included: the R6/2 mouse model at 4, 9, and 12 weeks of age (n > 6) and their C57BL/6J littermates (n > 6); the R6/1 mouse model at 2, 6, and 8 months of age (n > 6) and their C57BL/6J littermates (n > 6); and the *Hdh*Q150 homozygote mice at 4, 14, and 23 months of age (n = 5), compared to their C57BL/6J littermates (n = 5). All animals had unlimited access to water and breeding chow (Special Diet Services, Witham, UK), and housing conditions and environmental enrichment were as described previously [25].

### 2.2. RNA Extraction and Taqman Real-Time PCR Expression Analysis

Total RNA from eye tissues was extracted with the mini-RNA kit (Qiagen, Manchester, UK), according to the manufacturer’s instructions. The reverse transcription reaction was performed using MMLV superscript reverse transcriptase (Invitrogen, Carlsbad, CA, USA) and random hexamers (Sigma, New York, NY, USA), as described in an earlier study [25,26]. All Taqman qPCR reactions were performed with a LightCycler^®^ 480 Instrument (Roche, Welwyn Garden City, UK), as described previously [17]. Estimation of mRNA copy number was determined in triplicate for each RNA sample by comparison with the geometric mean of three endogenous housekeeping genes (Primer Design, London, UK), as described [17]. Stable housekeeping genes for qPCR profiling of eye tissues for HD mouse models at specific time points were determined using the Primer Design *geNorm™ Housekeeping Gene Selection Mouse Kit with PerfectProbe™* software, as described previously [26], which contains a panel of genes: *Atcb* (Actin, beta, cytoplasmic, 11461), *Gapdh* (Glyceraldehyde-3-phosphate dehydrogenase, 14433), *Ubc* (Ubiquitin C, 22190), *B2m*, (Beta-2-microglobulin, 12010), *Ywhaz* (Phospholipase A2, 22631), *Rpl13a* (Ribosomal protein L13a, 22121), *Canx* (Calnexin, 12330), *Cyc1* (Cytochrome c-1, 66445), *Sdha* (Succinate dehydrogenase complex, subunit A, 66945), *18S* (18S rRNA, 19791), *Eif4A2* (Eukaryotic translation initiation factor 4A2, 13682), and *Atp5b* (ATP synthase subunit, 11947). The following Taq-man assays for selected genes of interest, from Thermo Fisher Scientific, were used in this study: *Fabp7* (Mm00445225_m1), *Arr3* (Mm00504628_m1), *Opn1mw* (Mm00433560_m1), *Opn1sw* (Mm00432058_m1), *Gnat2* (Mm00492394_m1), *Pfkfb2* (Mm00435575_m1), and *C1ql3* (Mm00655312_m1).

### 2.3. RNA Isolation for Array Analysis

The total RNA was extracted using TRI Reagent (Sigma-Aldrich, St. Louis, MO, USA) and purified on columns (NucleoSpin Total RNA Isolation, Qiagen GmbH, Hilden, Germany). The amount of total RNA was determined by optical density at 260 nm and its purity was estimated by 260/280 nm absorption ratio (higher than 1.8 required) (NanoDrop spectrophotometer, Thermo Scientific, Waltham, MA, USA). The RNA integrity and quality were also verified by Bioanalyser 2100 (Agilent Technologies, Inc., Santa Clara, CA, USA). The resulting RNA integrity numbers (RINs) were between 8.5 and 10 with an average of 9.2. Each sample was diluted to the RNA concentration of 100 ng/μL. From each RNA sample, 100 ng of RNA was taken for microarray experiments.

### 2.4. Microarray Expression Study

The microarray study was carried out according to previously described protocols [27,28,29]. The isolated RNA was pooled into fifteen samples, representing wild type littermates (n = 3) and R6/1 mice (n = 4), at the symptomatic stage of 6 months of age; wild type littermates (n = 4) and R6/2 mice (n = 4), at the symptomatic stage of 9 weeks of age. The total RNA (100 ng) from each sample was subjected to two rounds of sense cDNA amplification, biotin labelling, and fragmentation according to the manufacturer’s protocol. (GeneChip^®^ WT Plus Reagent Kit, Affymetrix, Santa Clara, CA, USA). Biotin-labelled fragments of cDNA (5.5 μg) were hybridised to the Affymetrix^®^ Mouse Gene 2.1 ST Array Strip (45 C/20 h). After hybridisation, array strips were washed and stained by the Fluidics Station of a Gene Atlas System (Affymetrix). Next, the array strips were scanned by the Imaging Station from a Gene Atlas System. Preliminary analysis of the scanned microarrays was performed using Affymetrix Gene Atlas TM Operating Software (Affymetrix, Santa Clara, CA, USA). The quality of gene expression data was checked according to quality control criteria provided by the software. The obtained CEL files were imported into downstream data analysis.

### 2.5. Microarray Data Analysis

All analyses were performed using BioConductor software with the relevant Bioconductor libraries, based on the statistical R programming language (v4.1.2; R Core Team 2021). The Robust Multiarray Average (RMA) normalization algorithm implemented in the “Affy” library was used for normalization, background correction, and calculation of the expression values for all examined genes [30]. Biological annotation was taken from the BioConductor “mogene21sttranscriptcluster.db” package, where the annotated data frame object was merged with the normalized data set, leading to a complete gene data table [31]. The set of genes with low variance was removed by a variance-based filtering function from the “genefilter” library [32]. Differential expression and statistical assessment were determined by applying the linear models for microarray data implemented in the “limma” library [33]. The selection criteria for a significantly changed gene expression were based on *p*-value < 0.05 with 20% of false discovery rate (FDR) correction. The result of this selection was presented as a volcano plot, showing the total number of up- and down-regulated genes. Principal component analysis (PCA) of the filtered data set was performed and visualized using the “factoextra” library [34].

Raw Data files were also deposited in the Gene Expression Omnibus (GEO) repository at the National Center for Biotechnology Information (http://www.ncbi.nlm.nih.gov/geo/ accessed on 25 March 2022) under the GEO accession number: GSE 199335.

### 2.6. Assignment of Differentially Expressed Genes to Relevant Gene Ontology (GO) Terms

The whole set of differentially expressed genes (DEGs) were subjected to functional annotation and clustering using the DAVID (Database for Annotation, Visualization, and Integrated Discovery) bioinformatics tool [35]. All gene IDs of differentially expressed genes were uploaded to DAVID by the “RDAVIDWebService” BioConductor library [36], where DEGs were assigned to relevant GO terms, with a subsequent selection of significantly enriched GO terms from the GO BP FAT database. The *p*-values of selected GO terms were corrected using Benjamini–Hochberg correction, described as adjusted *p*-values [37]. Differentially expressed genes from each comparison were visualised as a heatmap using the “ComplexHeatmap” library [38], where the 9 most significantly-enriched ontological groups (lowest adjusted *p*-values) were shown as dots. To find the common DEGs in two symptomatic HD mouse models, namely R61 at 6 months of age and R6/2 at 9 weeks of age, the “ggvenn” library was used [39]. List of common DEGs ENTREZ IDs were functionally analyzed using the Metascape tool. Metascape combines functional enrichment, interactome analysis, gene annotation, and membership search to leverage over 40 independent knowledgebases [40]. The ontological terms that were significantly regulated were visualised as a bar plot. The association of individual genes with relevant ontological terms was presented using Cytoscape (v. 3.7.2) software [41].

## 3. Results

To date, our knowledge of potential biomarkers that can be linked to the eye pathological remodeling in Huntington’s disease is very limited. Hence, in this study, we aimed to establish the transcriptional signature related to the pathology of the eye tissue, using an Affymetrix array and qPCR assays in various HD mouse models. To test the hypothesis that mutant HTT leads to transcriptional remodeling of the eye transcriptome, we used two types of well-established HD mouse models. These included so called fragment models that are transgenic for a mutated N-terminal exon 1 HTT fragment, namely R6/2 and R6/1 [4], and the full length model *Hdh*Q150, which has an expanded CAG repeat knocked-in to the mouse huntingtin gene (*Htt*) [42]. We performed our analysis during the onset of HD symptoms in all three HD mouse models, representing different stages of disease progression, as established previously [16,24,25]: the pre-symptomatic stage (R6/2 at 4 weeks of age; R6/1 at 2 months of age; *Hdh*Q150 at 4 months of age); the symptomatic stage (R6/2 at 9 weeks of age; R6/1 at 6 months of age; *Hdh*Q150 at 14 months of age); and the end-stage (R6/2 at 12 weeks of age; R6/1 at 8 months of age; *Hdh*Q150 at 23 months of age).

**Figure 1:**
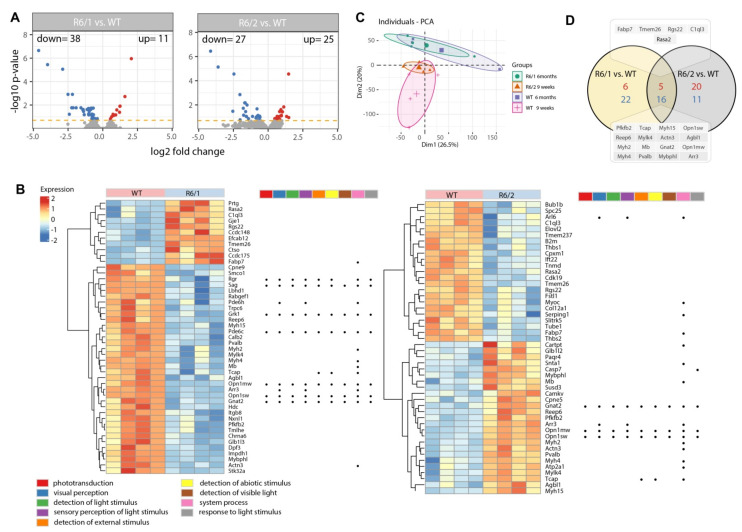
Transcriptome profiling in the eyes of symptomatic Huntington’s Disease (HD) mouse models. Nine weeks old R6/2 mice and 6 months old R6/1 mice were analyzed. (**A**) Volcano plots where each dot represents the average normalized expression of a single gene. Orange dashed lines represent cut-off values (*p* < 0.05 with 20% false discovery rate correction). Blue dots are down-regulated genes, while red dots are up-regulated. (**B**) Heatmap with hierarchic clustering of differentially expressed genes. Genes belonging to the significantly enriched ontological group with the strongest effects (lowest adjusted *p*-value) are shown as dots. (**C**) Principal component analysis (PCA) plots showing the first two principal components of the filtered microarray data set. (**D**) Venn diagram displaying different and common DEGs regulated in the R6/1 vs. WT 6 months old mice and R6/2 vs. WT 9 weeks old mice. Symbols of common genes are shown.

We began our comprehensive analysis of the eye transcriptome by employing Affymetrix array analysis in two HD mouse models at the symptomatic stage: R6/1 at 6 months of age and R6/2 at 9 weeks of age. The transcriptome of HD mouse models was compared to age-matched wild type controls. Volcano plots (Figure 1A) represent overall transcriptional changes in the HD eye tissues; here we found 38 down-regulated and 11 up-regulated genes in the symptomatic R6/1 mice, while the symptomatic R6/2 mouse transcriptome showed 27 genes to be down-regulated and 25 up-regulated. We used stringent cut-off values (*p* < 0.05 with 20% false discovery rate correction) for our analysis. The list of significantly deregulated genes is presented as a heatmap for each investigated mouse model separately (Figure 1B). In addition, Principal Component Analysis (PCA) plots, showing the first two principal components of the filtered microarray data set, are shown (Figure 1C). The PCA analysis showed a better separation of samples based on age with less apparent differences between wild type and HD samples. In order to find a common set of differentially expressed genes in these two symptomatic mouse models, we used the “ggvenn” library and plotted those common genes representing HD onset as a Venn diagram (Figure 1D). We found 5 genes to be significantly up-regulated and 16 to be significantly down-regulated in both HD mouse models.

**Figure 2:**
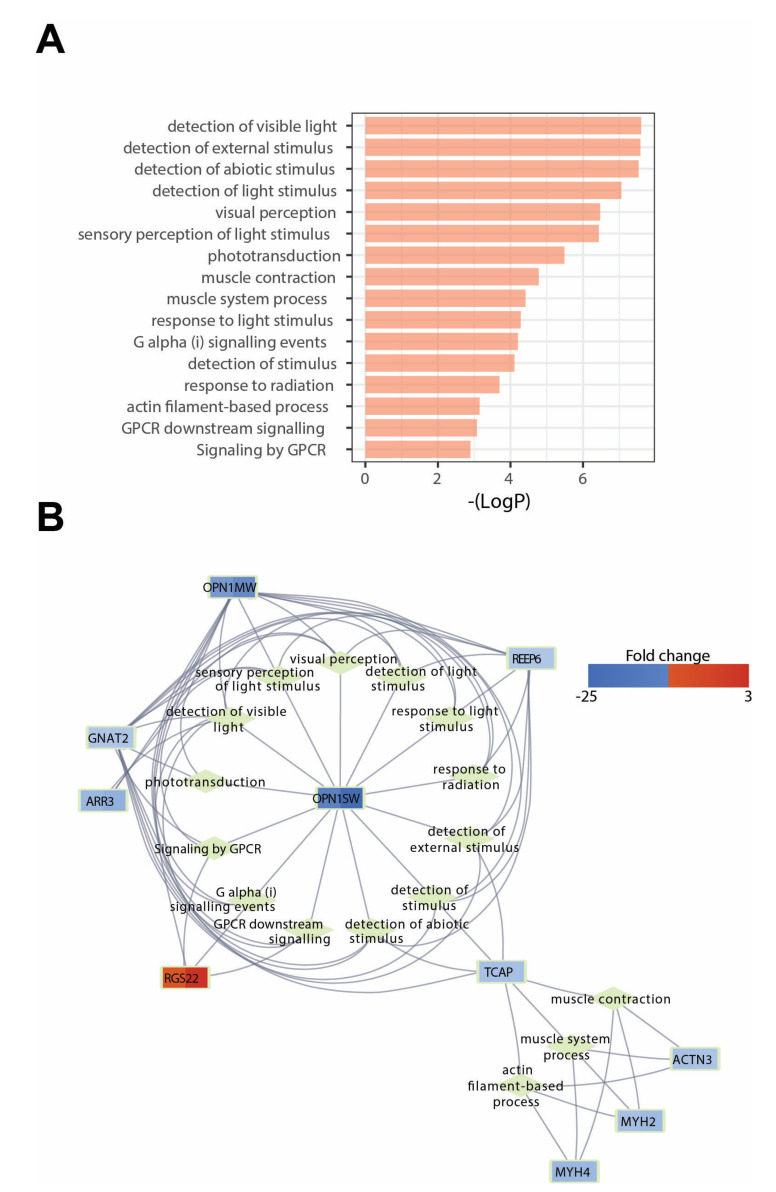
Ontological group regulated by common differentially expressed genes (DEGs) in two symptomatic HD mouse models: R6/2 vs. WT at 9 weeks of age and R6/1 vs. WT at 6 months of age. (**A**) Significantly enriched GO terms by common DEGs by Metascape analysis. (**B**) Interaction network between common DEGs and enriched GO terms. Fold change values for individual genes are marked by a color scale.

Next, we performed Metascape analysis to identify significantly enriched GO terms based on common DEGs (Figure 2A). As a result, we identified a number of ontological groups significantly deregulated in both symptomatic HD mouse models, including those related to the detection of visible light, visual perception, phototransduction, and the detection of external stimuli, as well as muscle contraction and muscle system processes, to name but a few. These deregulated processes mirrored our further interaction network analysis based on common DEGs and enriched GO terms (Figure 2B), where we found a number of deregulated networks linked to either to phototransduction, the detection of visible light, muscle contraction, or muscle processes. In summary, the transcriptional changes in the eye tissue of two symptomatic HD mouse models strongly indicated that genes related to retina remodelling and muscle contractions are typical features of the HD eye transcriptome.

The array analysis gave us vital information about overall transcriptome changes in HD eye tissue. Hence, in order to identify biomarkers that can be potentially used to monitor disease progression, we employed qPCR analysis—an assay that allows us to precisely quantify deregulated genes in relation to the disease stage. For further analysis, we focused on differentially expressed genes related to retina remodelling only, since it is already well established that mutant HTT also leads to HD-related skeletal muscle atrophy [16,18,43]. This analysis was additionally performed on the full-length HD mouse model, *Hdh*Q150. We selected three distinct time points for each mouse model, representing the advance of the disease progression: the pre-symptomatic stage, where there are no apparent neurological and behavioral abnormalities; the symptomatic stage, where they are significant behavioral and molecular changes; and the end-stage, representing a severe phenotype with a full spectrum of pathological abnormalities, as we previously described in these HD mouse models [17,24,25].

It is well established that HD is characterised by large transcriptome abnormalities, therefore, we performed a study to identify suitable controls or reference genes. These had to be suitable for use in the expression analysis of eye tissues in the three HD mouse models, at the indicated time points. For this purpose, we used the geNorm™ Housekeeping Gene Selection Mouse Kit, and associated software, as previously described [17,26]. Thus, we identified the three most stably-expressed genes in the eye tissue (Figure S1). This assay was critical for our relative quantification method, which is based on the geometric mean of three selected reference genes for normalization, to accurately determine gene expression levels in WT and HD eye tissue, at specific time points.

Based on our array analysis, we selected a number of differentially expressed genes linked to retina remodeling, based on the interaction network analysis, to be further validated by quantitative PCR. This was carried out in all three HD mouse models at specific time points linked to the HD progression.

**Figure 3:**
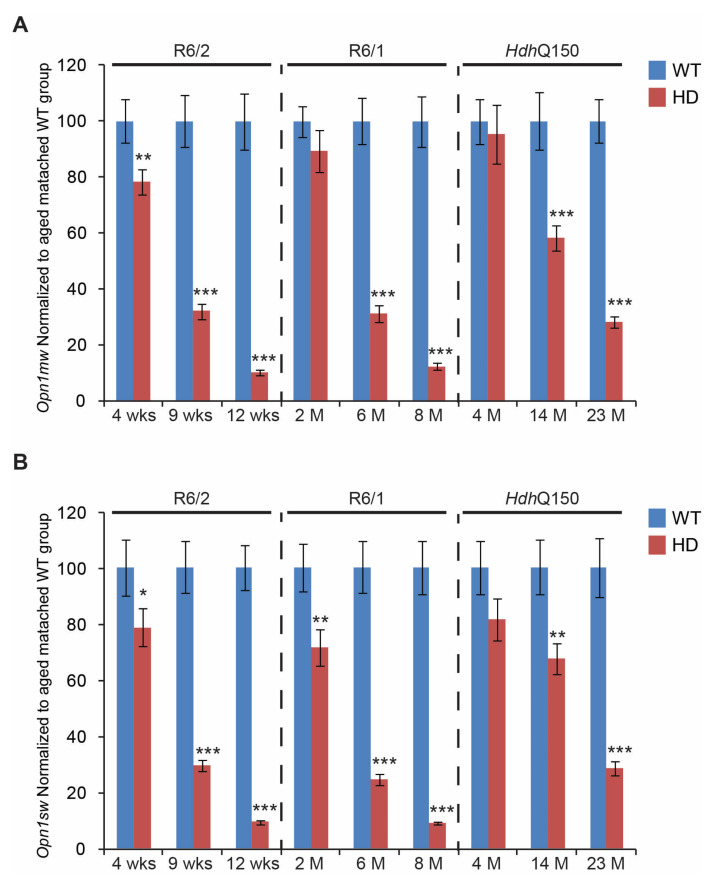
Transcriptional alterations of two *Opsin* genes involved in normal color vision. Transcripts of (**A**) *Opn1mw* (Opsin 1: cone pigments, medium-wave-sensitive)*,* (**B**) *Opn1sw* (Opsin 1: cone pigments, short-wave-sensitive), were assessed in the eye of three HD mouse models at: pre-symptomatic stage (4 weeks R6/2 mice, 2 months R6/1, mice and 4 months *Hdh*Q150 mice); symptomatic stage (9 weeks R6/2 mice, 6 months R6/1 mice, and 14 months *Hdh*Q150 mice), and end-stage (12 weeks R6/2 mice, 8 months R6/1 mice, and 23 months *Hdh*Q150 mice). All Taqman qPCR values were normalized to the geometric mean of three housekeeping genes, as indicated in the Materials and Methods section. Error bars are ± SEM (n = 6). SEM represents biological replicates. Student’s *t*-test: * *p* < 0.05, ** *p* < 0.01; *** *p* < 0.001.

**Figure 4:**
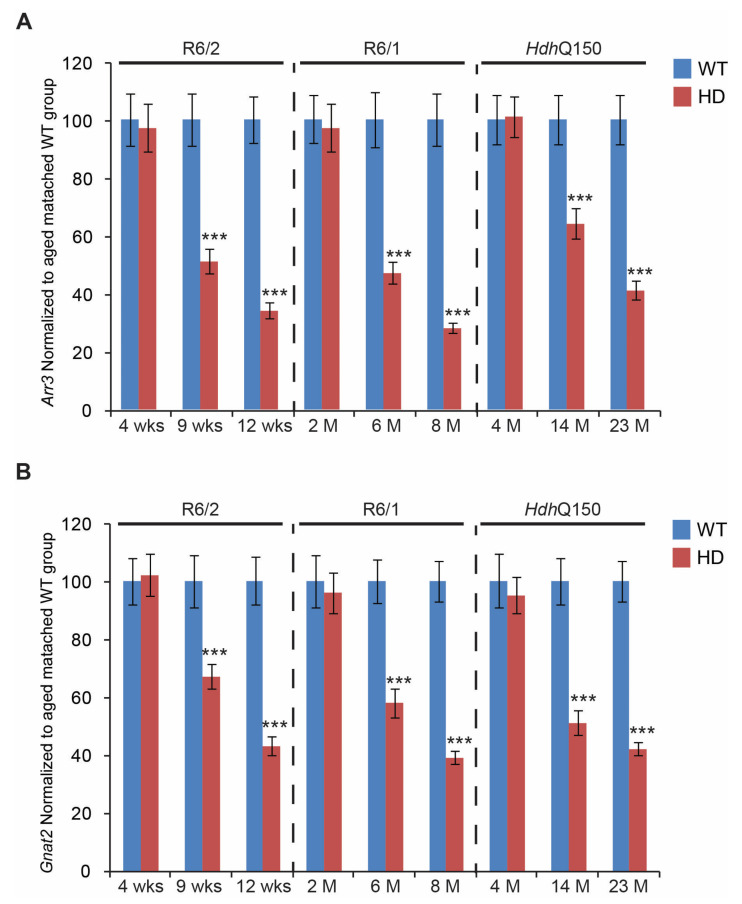
Transcriptional reductions of cone-specific genes. Transcripts of (**A**) *Arr3* (Arrestin 3, retinal)*,* (**B**) *Gnat2* (G protein subunit alpha transducin 2), were assessed in the eyes of three HD mouse models at: pre-symptomatic stage (4 weeks R6/2 mice, 2 months R6/1 mice, and 4 months *Hdh*Q150 mice); symptomatic stage (9 weeks R6/2 mice, 6 months R6/1 mice, and 14 months *Hdh*Q150 mice), and end-stage (12 weeks R6/2 mice, 8 months R6/1 mice, and 23 months *Hdh*Q150 mice). All Taqman qPCR values were normalized to the geometric mean of three housekeeping genes, as indicated in the Material and Methods section. Error bars are ± SEM (n = 6). SEM represents biological replicates. Student’s *t*-test: *** *p* < 0.001.

First, we found that the transcript levels of two opsin genes (involved in normal colour vision) were significantly down-regulated. *Opn1mw* (Opsin 1: cone pigments, medium-wave-sensitive) (Figure 3A) was significantly reduced in all three HD mouse models. In fact, a nearly 80% reduction of *Opn1mw* was found at the end-stage time point in R6/2, R6/1, and *Hdh*Q150 mouse models. Interestingly, the *Opn1mw* transcripts were already significantly reduced at the pre-symptomatic stage in the eye tissue of R6/2 mice. Transcript levels of *Opn1sw* (Opsin 1: cone pigments, short-wave-sensitive) were similarly severely down-regulated by approximately 80%, in R6/2, R6/1, and *Hdh*Q150 mouse models at end-stage (Figure 3B). Interestingly, *Opn1sw* mRNA was also significantly reduced at the pre-symptomatic stage in the R6/1 and R6/2 mice, while there was a trend towards reduction in the *Hdh*Q150 mice. Subsequently, we validated the transcriptional changes of cone-specific genes such as *Arr3* (Arrestin 3, retinal) and *Gnat2* (G protein subunit alpha transducin 2). We found that *Arr3* mRNA levels were unchanged at the pre-symptomatic stage of R6/1, R6/2, and *Hdh*Q150 mouse models, while there was a significant reduction, on average by 60%, of *Arr3* transcripts at the symptomatic and end-stages, in all three HD mouse models (Figure 4A). We also found a similar expression profile of the *Gnat2* mRNA (Figure 4B), whose transcripts were down-regulated significantly in all HD mouse models, at symptomatic and end-stages. Both *Arr3* and *Gnat2* transcripts were unchanged at the pre-symptomatic stage of R6/1, R6/2, and *Hdh*Q150 mouse models.

**Figure 5:**
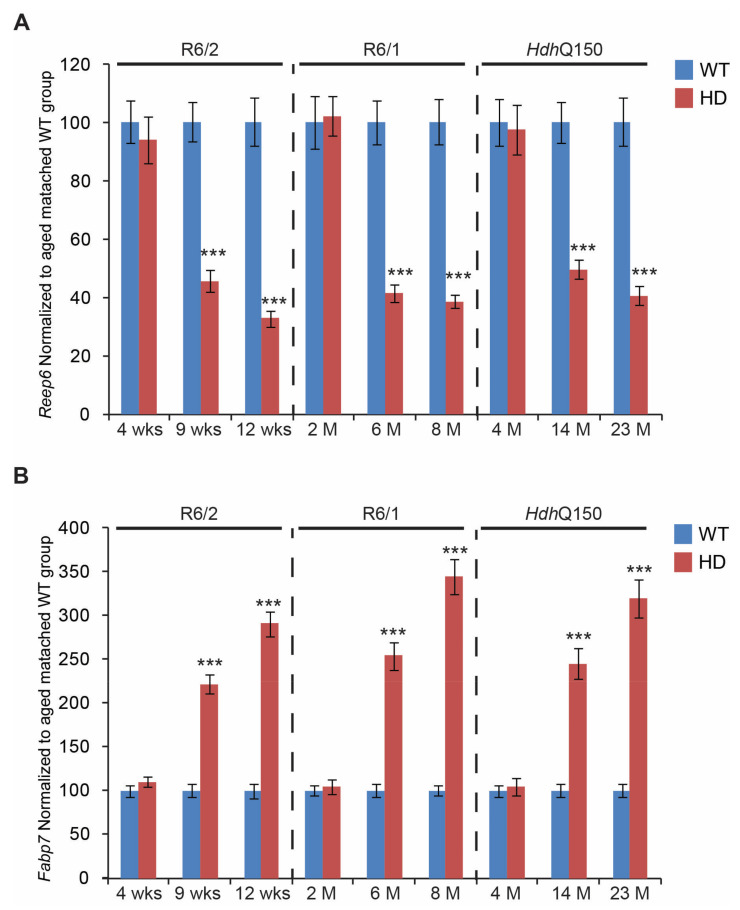
Transcriptional alterations of genes linked to retina degeneration. Transcripts of (**A**) *Reep6* (Receptor expression enhancing protein 6) and (**B**) *Fabp7* (fatty acid binding protein 7, brain), were assessed in the eyes of three HD mouse models at: pre-symptomatic stage (4 weeks R6/2 mice, 2 months R6/1 mice, and 4 months *Hdh*Q150 mice); symptomatic stage (9 weeks R6/2 mice, 6 months R6/1 mice, and 14 months *Hdh*Q150 mice), and end-stage (12 weeks R6/2 mice, 8 months R6/1 mice, and 23 months *Hdh*Q150 mice). All Taqman qPCR values were normalized to the geometric mean of three housekeeping genes, as indicated in the Materials and Methods section. Error bars are ± SEM (n = 6). SEM represents biological replicates. Student’s *t*-test: *** *p* < 0.001.

**Figure 6:**
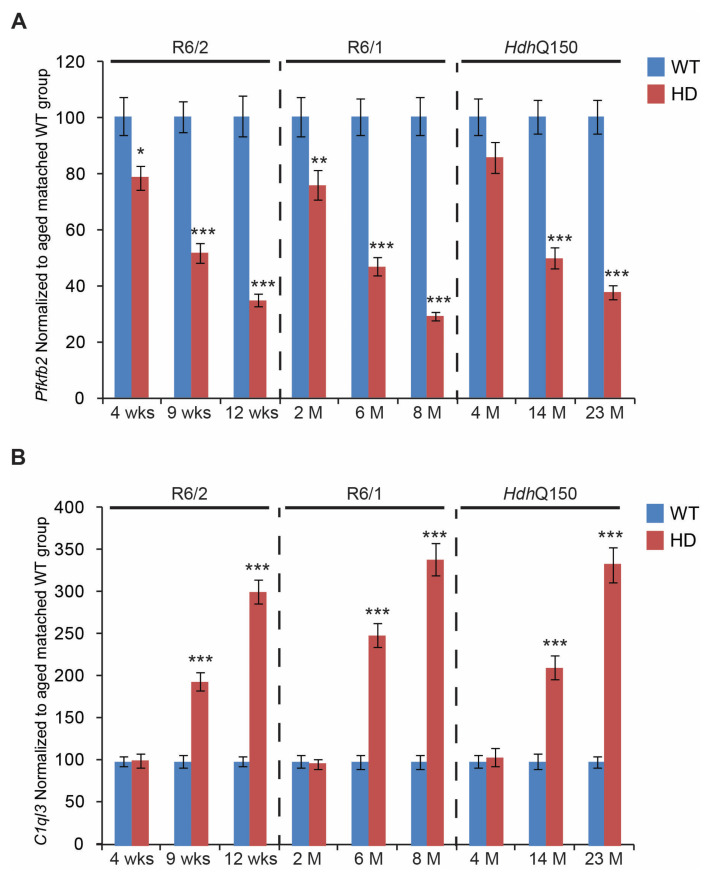
Transcriptional alterations of genes involved in energy homeostasis. Transcripts of (**A**) *Pfkfb2* (6-phosphofructo-2-kinase/fructose-2,6-biphosphatase2) and (**B**) *C1ql3* (Complement Component1, Q Sup-component-Like 3), were assessed in the eyes of three HD mouse models at: pre-symptomatic stage (4 weeks R6/2 mice, 2 months R6/1 mice, and 4 months *Hdh*Q150 mice); symptomatic stage (9 weeks R6/2 mice, 6 months R6/1 mice, and 14 months *Hdh*Q150 mice), and end-stage (12 weeks R6/2 mice, 8 months R6/1 mice, and 23 months *Hdh*Q150 mice). All Taqman qPCR values were normalized to the geometric mean of three housekeeping genes, as indicated in the Material and Methods section. Error bars are ± SEM (n = 6). SEM represents biological replicates. Student’s *t*-test: * *p* < 0.05, ** *p* < 0.01; *** *p* < 0.001.

We next assessed the transcriptional signatures of genes linked to retinal degeneration in some models of retinopathy. We found that *Reep6* (Receptor expression enhancing protein 6) transcripts were significantly down-regulated, by approximately 50%, in all HD mouse models but only at symptomatic and end-stages (Figure 5A). In contrast, there was a significant up-regulation of *Fabp7* (fatty acid binding protein 7, brain) mRNA, up to three-fold on average, in R6/1, R6/2, and *Hdh*Q150 mouse models at the symptomatic and end-stages (Figure 5B). Similarly to *Reep6* transcripts, there was no deregulation of *Fabp7* mRNA at the pre-symptomatic stage, in all HD mouse models. Our array analysis also identified two commonly deregulated genes in symptomatic R6/1 and R6/2 mice that are involved in energy homeostasis. Transcript levels of *Pfkfb2* (6-phosphofructo-2-kinase/fructose-2,6-biphosphatase2) were significantly down-regulated (on average by 60%) in all three HD mouse models, at both symptomatic and end-stages (Figure 6A). *Pfkfb2* mRNA was also found to be significantly down-regulated at the pre-symptomatic stage in the R6/2 mouse model, however, it remained unchanged in the two other mouse models (R6/1 and *Hdh*Q150). There was significant up-regulation, up to three-fold on average, of *C1ql3* (Complement Component1, Q Sup-component-Like 3) transcripts, in all HD mouse models, but only at the symptomatic and end-stages of the disease (Figure 6B). R6/1, R6/2, and *Hdh*Q150 mice did not display any alterations in *C1ql3* transcript levels at the pre-symptomatic stage.

In summary, we found a number of genes significantly deregulated, mainly at the symptomatic and end-stage, but more importantly their expression profile was very similar across all HD mouse models used in this study. Interestingly, we also found a couple of genes to be significantly deregulated at the pre-symptomatic stage, which might suggest that there is a signature of retinal pathological remodelling prior to central nervous degeneration, at the transcriptome level.

## 4. Discussion

Huntington’s disease belongs to a family of neurodegenerative disorders that are mainly characterised by a progressive structural and functional malfunction of the central nervous system (CNS) and peripheral nervous system (PNS) [6]. Extensive studies of pathological remodeling in many peripheral tissues (for a review see [18,43,44]) revealed that HD affects a number of organs and can be defined as a multi-system disorder [10]. It is still uncertain, however, whether those peripheral tissues are affected only indirectly, primarily via the CNS degeneration, or whether they are affected by the intrinsic intracellular effects of mutant HTT, which is expressed in virtually every cell type (for a review see [11,15]). One of the key molecular pathological features of HD is the accumulation of toxic mutant HTT aggregates during disease progression, not only in the CNS but also in other peripheral tissues [8,45]. Such mutant HTT aggregates have been also detected in the neuronal layers of the retina in a HD mouse model [20], consequently leading to a pathological remodeling of the retina at the molecular and physiological levels [20,21]. It is also well established that HD cells and tissues undergo significant deregulation of the transcriptome in the CNS but also in other peripheral organs. Intriguingly, some of those changes appear to occur earlier in the peripheral tissues, e.g., in the heart [13,15]. Since there was no previously available literature about the ocular transcriptome in HD, in this study we aimed to provide a broad overview of transcriptional deregulation of the eye tissue, in three symptomatic HD mouse models, using the approach of Affymetrix arrays. Most importantly, two of these mouse models develop full spectra of behavioural, neurological, and molecular features of HD, but within different time frames. Specifically, R6/2 mice reach the end-stage of the disease within 12 weeks of age, while the second mouse model, R6/1, develops the same set of symptoms within 8 months [17,25]. Our comparative array analysis was performed at the symptomatic stage of the R6/2 and R6/1 mice: at 9 weeks and 6 months of age, respectively. We found significant transcriptional changes in both validated HD mouse models, with 38 down-regulated and 11 up-regulated genes in the symptomatic R6/1 mice. Meanwhile, symptomatic R6/2 mouse transcriptomes showed 27 genes to be down-regulated and 25 to be up-regulated. Since our main aim in this study was to identify a robust panel of biomarkers that are common between various types of HD mouse models, we performed an analysis using “ggvenn library” and found 5 such up-regulated transcripts and 16 down-regulated ones. Furthermore, our Metascape analysis revealed a number of ontological groups, linked to the detection of visible light, visual perception, phototransduction, and the detection of external stimuli. This profile of consistently deregulated genes mirrors known pathological retinal pathology, described in the same HD mouse models used in this study [20,21]. In addition, our study identified a significant number of deregulated genes representing skeletal muscle pathological remodeling, which has already been described in specific muscle types [16,17]. Next, we were interested in validating the panel of robustly changed genes by the well-established quantitative PCR method. These assays could be applied during the disease progression at pre-symptomatic, symptomatic, and the end-stage of HD, in three well-established HD mouse models: R6/2, R6/1, and *Hdh*Q150. In order to extend the application of our newly identified biomarkers, we also added knock-in *Hdh*Q150 mice to the analysis. This model is representative of a full length HD gene insertion, and is relatively mild in phenotype, with the end-stage time point at 23 months of age [24].

Our qPCR analysis revealed that a group of Opsin transcripts, mainly *Opn1mw* and *Opn1sw*, were severely down-regulated in all three HD mouse models, by approximately 80% at the end-stage of disease. This is in line with previous studies in the R6/1 mouse model, where the loss of cone opsins was described as being progressive, and leading to a nearly complete absence of the protein at the end-stage [21]. The *Opn1mw* gene is essential for normal colour vision and it has been shown that its transactivation delays retinal degeneration and improves retinal function in the heterozygous rhodopsin-deficient (Rho+/−) RP mouse model [46]. The *Opn1sw* gene provides instructions for making an opsin pigment that is more sensitive to light in the blue/violet part of the visible spectrum [47]. In addition, our analysis found two additional cone specific genes, *Arr3* and *Gnat2,* to be significantly down-regulated at the symptomatic and end-stages, in all three HD mouse models. Those two genes were not previously linked to HD-related retinopathy, however, *Arr3* mRNA has been also found to be down-regulated in channel-deficient retinas or mouse models of cone degeneration [48]. Interestingly, at least 10 identified mutations in the *GNAT2* gene have been found to be a source of the vision disorder achromatopsia [49]. A pre-clinical study also found that *Gnat2* deficient mice showed a complete loss of cone-driven waves, based on ERG recordings [50]. Hence, one might conclude that HD-related retinopathy might display some characteristics of achromatopsia.

Moreover, we found transcriptional deregulation of two other genes, *Reep6* and *Fabp7,* which have been linked directly to retinal degeneration. It has been shown that Reep6 knockout mice display a rod degeneration phenotype [51]. In addition, several mutations have been identified in the *REEP6* gene that are linked to the development of an autosomal recessive disease called *Retinitis pigmentosa* [52]. In HD mouse models, this gene was also found here to be significantly down-regulated at the symptomatic and end-stages, in the R6/1, R6/2, and *Hdh*Q150 mice. Interestingly, rescuing the Reep6 mutant phenotype via gene replacement therapy showed significant improvements in the photoresponse and preserved photoreceptor cells [53]. In contrast, the *Fabp7* gene has been found to be significantly up-regulated in HD mouse models. It has been shown that Fatty acid binding protein 7 is expressed in the inner nuclear layer, outer plexiform layer, and photoreceptor inner segments; *Fabp7* deficient mice have significantly decreased retinal venular calibre and retinal arteriolar fractal dimension, as well as significantly increased areas of fluorescein leakage in the retina [54]. Since the HD mouse models displayed a significant up-regulation of this gene, this could be attributed to a possible pro-inflammatory response because it has been shown that FABP7 overexpression leads to an NF-κB-driven pro-inflammatory response in astrocytes [55]. Hence, it can be concluded that Fabp7 is a part of the immunological response in HD mice models.

Finally, we identified changes in gene expression that are linked to energy homeostasis. The *Pfkfb2* mRNA was significantly down-regulated even at the pre-symptomatic stage of the two HD mouse models, R6/1 and R6/2. This enzyme is a member of a small gene family encoding four PFKFB isoenzymes. They regulate the formation and degradation of fructose-2,6-biphosphate (F-2,6-P2), a signalling molecule that controls glycolysis by regulating phosphofructokinase-1 (PFK-1) activity [56]. Consequently, the knock-down of PFKFB led to the inhibition of glycolysis [57] and blocked glucose uptake and lipogenesis in prostate cancer cells [58]. In contrast, we found that *C1ql3* mRNA was significantly up-regulated (by up to three-fold) in all three HD mouse models. This gene function was initially described in adipose and pancreatic β-islet cells, with apparent functions in energy metabolism, glucose uptake, and insulin secretion [59,60,61]. In summary, mutant HTT results in the deregulation of the ocular transcriptome, leading to a pathological remodelling of the retina in both fragment mouse models of HD, such as R6/2 and R6/1, as well as in the full length *Hdh*Q150 mouse model.

In summary, we report the first transcriptional signature that represents changes in the pathological remodeling of the retina in three well-established HD mouse models. Although the CNS in HD is still believed to be the main source of major behavioural and neurological malfunctions, it should also be emphasized that the ocular organ, which is interconnected to the brain, may also play a pivotal role in the CNS dysfunction. Retinal progressive degeneration has been already described in other neurodegenerative disorders such as Alzheimer’s disease [62] and Parkinson’s disease [63]. Importantly, we identified a set of genes whose expression is already altered at the pre-symptomatic stage, prior to pathological features occurring in the CNS. In addition, two altered genes identified in this study have been previously described as a source of two other vision disorders: achromatopsia and *Retinitis pigmentosa.* We believe that the genes identified here might provide a useful set of HD biomarkers, both for tracking disease progression and for tracking the effects of new therapies that are under development.

## Data Availability

Raw Data files are available from the Gene Expression Omnibus (GEO) repository at the National Center for Biotechnology Information (http://www.ncbi.nlm.nih.gov/geo/, accessed on 25 March 2022) under the GEO accession number GSE 199335.

## Supplementary Materials

The following supporting information can be downloaded at: https://www.mdpi.com/article/10.3390/cells11101675/s1, Figure S1: Identification of reference genes for qPCR from the eye tissues of HD mouse models.
