# Supplementary material for: Identification of the Transcriptional Biomarkers Panel Linked to Pathological Remodelling of the Eye Tissues in Various HD Mouse Models
Source: Cells. 2022 May 18;11(10):1675. doi: 10.3390/cells11101675 (PMC9139483; doi:10.3390/cells11101675)
Supplement: Supplementary file 1 [file cells-11-01675-s001.zip › cells-1663960-supplementary.pdf]

SUPPLEMENTARY FIGURE

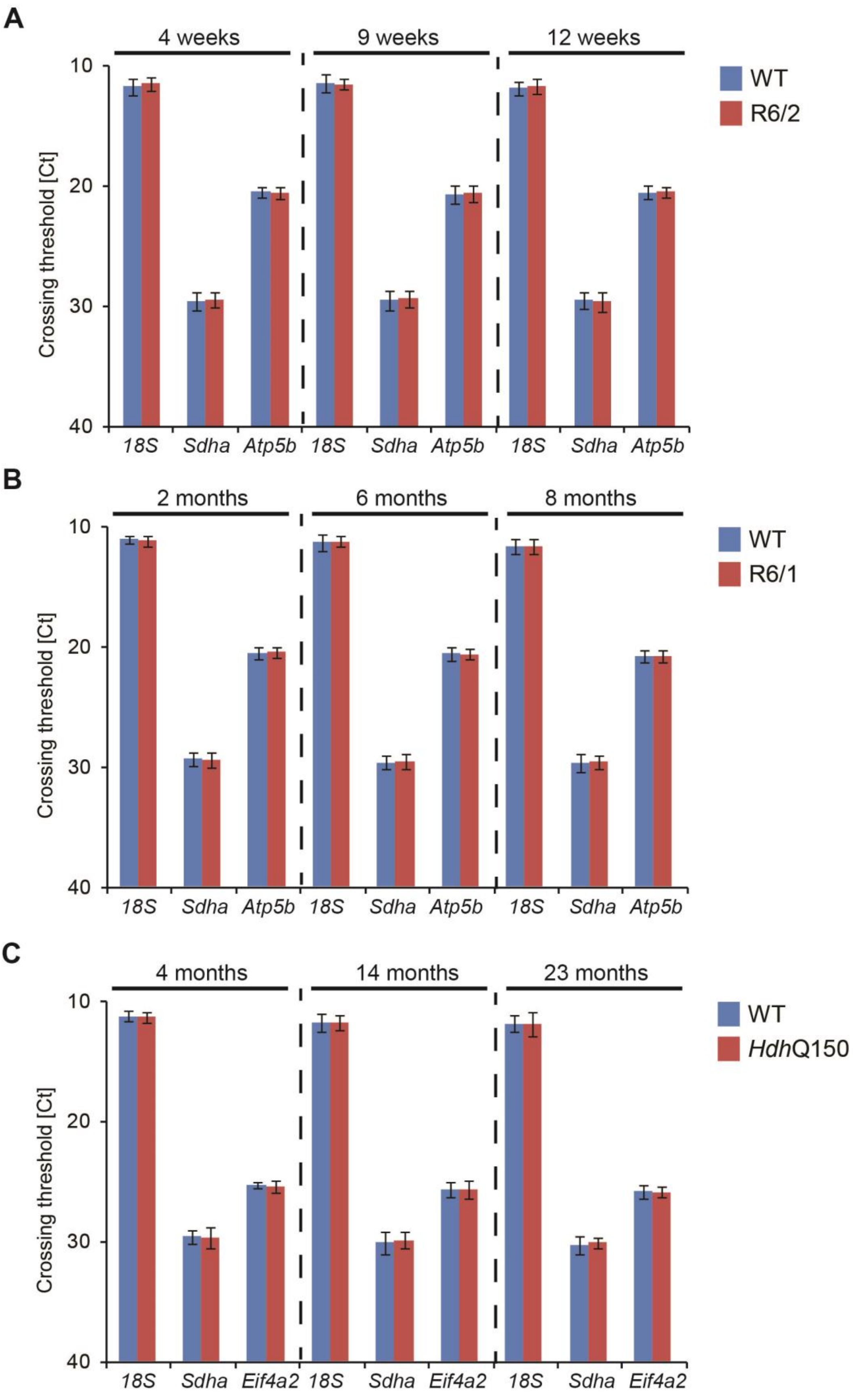

Figure S1. Identification of reference genes for qPCR from the eye tissues of HD mouse models. GeNorm analysis was used to identify optimal reference genes. Raw crossing threshold (Ct) data for a panel of three reference genes from the geNorm kit are shown (A) R6/2 mouse model at the age of 4 weeks, 9 weeks and 12 weeks, (B) R6/1 mouse model at the age of 2 months, 6 months and 8 months, (C) *Hdh*Q150 mouse model at the age of 4 months, 14 months and 23 months. *Sdha* (Succinate dehydrogenase complex, subunit A, 66945), *18S* (18S rRNA, 19791), *Eif4A2* (Eukaryotic translation initiation factor 4A2, 13682), *Atp5b* (ATP synthase subunit, 11947).
